# Host Cell Factors as Antiviral Targets in Arenavirus Infection

**DOI:** 10.3390/v4091569

**Published:** 2012-09-13

**Authors:** Florencia N. Linero, Claudia S. Sepúlveda, Federico Giovannoni, Viviana Castilla, Cybele C. García, Luis A. Scolaro, Elsa B. Damonte

**Affiliations:** Laboratorio de Virología, Departamento de Química Biológica, Facultad de Ciencias Exactas y Naturales, Universidad de Buenos Aires/IQUIBICEN (CONICET), Ciudad Universitaria, Pabellón 2, Piso 4, Buenos Aires 1428, Argentina; Email: flinero@qb.fcen.uba.ar (F.N.L.); claudia@qb.fcen.uba.ar (C.S.S.); fedegiova@gmail.com (F.G.); viviana@qb.fcen.uba.ar (V.C.); cygarcia@qb.fcen.uba.ar (C.C.G.); luisco@qb.fcen.uba.ar (L.A.S.)

**Keywords:** arenavirus, Junin virus, hemorrhagic fever, antiviral, host cell target

## Abstract

Among the members of the *Arenaviridae* family, Lassa virus and Junin virus generate periodic annual outbreaks of severe human hemorrhagic fever (HF) in endemic areas of West Africa and Argentina, respectively. Given the human health threat that arenaviruses represent and the lack of a specific and safe chemotherapy, the search for effective antiviral compounds is a continuous demanding effort. Since diverse host cell pathways and enzymes are used by RNA viruses to fulfill their replicative cycle, the targeting of a host process has turned an attractive antiviral approach in the last years for many unrelated virus types. This strategy has the additional benefit to reduce the serious challenge for therapy of RNA viruses to escape from drug effects through selection of resistant variants triggered by their high mutation rate. This article focuses on novel strategies to identify inhibitors for arenavirus therapy, analyzing the potential for antiviral developments of diverse host factors essential for virus infection.

## 1. Introduction

Arenaviruses have turned in recent years a serious and increasing challenge for human public health, particularly in the Americas and Africa. Among the members of the *Arenaviridae* family, Lassa virus (LASV) and Junin virus (JUNV) generate periodic annual outbreaks of severe human hemorrhagic fever (HF) in endemic areas of West Africa and Argentina, respectively. LASV is the most prevalent and dangerous arenavirus, causing over 300,000 cases of Lassa fever per year and between 5,000 and 10,000 deaths [1]. By contrast, the incidence and severity of Argentine HF produced by JUNV is considerably lower, with 100–1,000 notified cases per year and a case-fatality rate about 15% in the absence of treatment [2]. Besides the critical situation in endemic areas, the high frequency of international air travels has also contributed to the importation of arenavirus HF cases into several urban areas around the world [3]. In addition to these two pathogens that represent the main health threat in the family, there are also four recognized arenaviruses, Sabiá, Guanarito, Machupo and Chapare virus, able to produce very sporadic cases of HF in Brazil, Venezuela and Bolivia, respectively. The prototype world-wide distributed arenavirus lymphocytic choriomeningitis virus (LCMV) can also infect humans, generally resulting in an asymptomatic course or a mild febrile illness sometimes associated with aseptic meningitis. However, LCMV is of considerable concern in pediatrics in cases of congenital infection [4] and also for immunocompromised patients, as recently shown in cases of fatal LCMV infection acquired through transplantation [5]. Furthermore, a continuous and extended emergence of new agents tentatively included as new members of *Arenaviridae* has occurred in the last decade either from accidental human infections causing a severe disease or as result of a systematic screening for virus or genome presence in the natural rodent reservoirs [6].

In spite of the health burden depicted by arenavirus infections, no safe and effective chemotherapy is currently available allowing for consider these pathologies as neglected viral diseases. Antiviral therapies are limited to the use of immune convalescent plasma with defined doses of JUNV-neutralizing antibodies, recommended for Argentine HF patients [2], or the guanosine analog ribavirin (1-β-D-ribofuranosyl-1,2,4-triazole-3-carboxamide) (RIB), effective against Lassa fever by intravenous administration [7]. The use of these different treatments is a direct consequence of the different mechanism of protection associated to both arenavirus HF: protection in Argentine HF is based on induction of a strong humoral immune response [8] whereas in Lassa fever patient recovery is mainly associated to cell-mediated immune response with low antibody production [1]. However, several drawbacks are associated to both treatments: RIB is not efficient in advanced LASV infections and it can also induce adverse side effects such as thrombocytosis, anemia and birth defects [7,8,9,10] whereas plasma transfusion also is not effective in advanced cases and 10% of treated patients develop late neurological complications [2].

On this basis, there is an essential need for the development of novel therapeutic options against arenaviruses. Two mainly different approaches can be employed for viral chemotherapy development: the viral-target based approach, directed to block a virus encoded function, and the host-target based approach, aimed to inhibit any cellular function required for virus multiplication and/or pathogenesis. This article is focused on diverse cellular targets essential for virus infection and their perspectives for specific chemotherapy against arenaviruses.

## 2. Targeting Host Cell Factors: Advantages and Disadvantages

Since licensed antiviral drugs in current use against other viruses target viral proteins, they are usually virus-specific and are prone to induce the fast appearance of viral resistant mutants. Resistance to viral inhibitors is a particularly more serious problem for therapy of clinically important RNA viruses, given their high mutation rates [11,12]. An alternative to mitigate this problem could be the development of drugs that affect host factors required for completion of virus replication cycle rather than directly pathogen encoded-factors. There are a number of processes for virus multiplication within the infected cell that involve cellular pathways and enzymes which have proven to be attractive targets for chemotherapeutic intervention against several unrelated viruses [13,14,15,16]. This approach is expected to establish a high barrier against viral escape from inhibition since it is not expectable that individual viral mutations will compensate for the loss of a required host factor.

Besides the rare emergence of virus resistance, therapy by inhibiting cellular pathways provides a promising target for the development of broad-spectrum antivirals active against all the viruses of the same genus/family and even unrelated ones as many viruses may share a dependency on the same host function. Another advantage of cellular targets is the possibility to employ as antiviral agents the existing drugs licensed for other human disorders with defined safety-data profiles and available clinical-use histories, requiring only assessment for the new indirect use.

On the other hand, targeting host factors might result sometimes in cytotoxic or other undesirable side-effects. To minimize this problem, inhibition needs to be directed with accuracy. In this aspect, the application of a host-based antiviral strategy appears to be more suitable for treatment of infections caused by pathogens associated with acute disease in humans, like HF arenaviruses, since the treatment time is limited and possible collateral effects may be minimized.

An important limitation for evaluating antiviral strategies to combat HF arenaviruses is the requirement of BSL4 facilities to handle live infectious virus in the classical cytopathic effect/plaque reduction or virus yield inhibition assays. The development of reverse genetics systems for several members of the family, and recently in particular for JUNV [17,18] and LASV [19], has provided a very useful tool to discover and characterize antiviral drugs against arenaviruses, overcoming the biosafety restrictions since the production of infectious virus is not involved in these assays. In this context, retroviral pseudotypes bearing HF arenavirus glycoproteins and a luciferase reporter gene were used in high-throughput screening (HTS) of small molecule libraries for virus entry inhibitors [20,21]. A cell-based luciferase assay for rapid and quantitative evaluation of arenavirus budding inhibitors, suitable for HTS, was also developed in the last years [22] and it could be used in combination with siRNA-based screens against cellular genes illustrating the wide perspectives of these new technologies to improve and facilitate the identification of cellular targets for HF chemotherapy.

To further support the rationale behind this antiviral strategy it is necessary to fully understand the significance and involvement of virus-cell interactions for proper viral infection. Novel automated genomics and proteomics together with bioinformatics have provided the tools to advance in the study of virus-host interactome in diverse pathogens [23,24,25]. Although there is still a little application of these technologies in arenaviruses [26,27,28,29], numerous reports for cell-based inhibitory strategies have been reported in the last years. 

**Figure 1:**
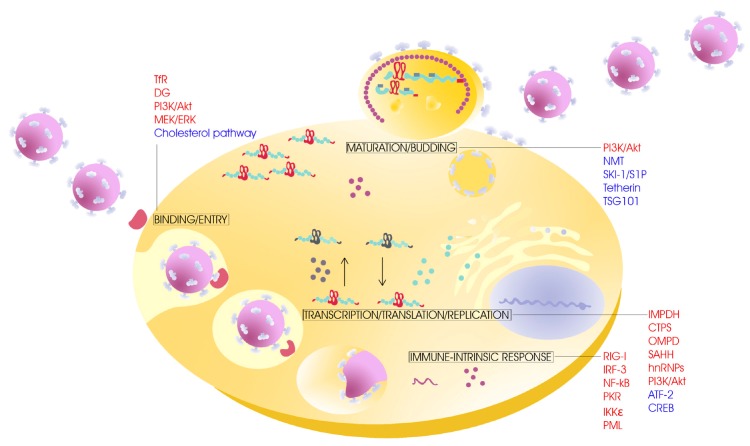
Main host factors involved in arenavirus multiplication analyzed as potential antiviral targets. In an infected cell the cellular factors discussed in this article (IMPDH: inosine monophosphate dehydrogenase; CTPS: cytosine triphosphate synthetase; SAHH: S-adenosylhomocysteine hydrolase; OMPD: orotidylic acid decarboxylase; hnRNPs: heterogeneous nuclear ribonucleoproteins; PI3K/Akt: phosphatidylinositol-3-kinase/protein kinase B; MEK/ERK:mitogen-activated protein kinase/extracellular signal-regulated kinase; IRF-3: interferon regulatory factor 3; IKKε: inhibitor of nuclear factor kB kinase–related kinase; NF-κB: nuclear factor kappa B; RIG-I: retinoic acid-inducible gene I protein; PKR: protein kinase R; PML: promyelocytic leukemia protein; DG: dystroglycan; TfR: transferrin receptor) are indicated in red; other factors not discussed here (TSG101: tumor supresor gene 101; NMT: *N*-myristoyltransferase; SKI-1/S1P: subtilisin kexin isozyme-1/site 1 proprotein convertase; ATF-2: activating transcription factor 2; CREB: cAMP response element-binding [30,31,32,33] are in blue.

The main host factors or processes involved in arenavirus multiplication that have been analyzed as potential antiviral targets *in vitro* are summarized in Figure 1. Recent studies performed with JUNV on particular antiviral cell-targeted approaches with interesting perspectives will be further discussed here.

## 3. Cellular Proteins Involved in RNA Synthesis or Processing

### 3.1. Metabolism of Nucleosides

In the context of cellular targets, one of the most explored to date is represented by cellular enzymes involved in the various metabolic pathways related to cell RNA synthesis and also engaged in virus RNA transcription and/or replication. Precisely RIB, the only antiviral in clinical use for HF caused by arenaviruses, is a prototypical example of this class of agents. 

RIB is a guanosine analog which once inside the cell is phosphorylated first to RIB-5’-monophosphate and then to RIB-5’-triphosphate. The primary target for this compound is supposed to be the cellular enzyme inosine monophosphate dehydrogenase (IMPDH) that converts IMP to xanthosine monophosphate, a limiting step for de novo intracellular synthesis of guanosine nucleotides [34,35]. The blockade of IMPDH by RIB-5’-monophosphate decreased the intracellular GTP pool with the consequent reduction of viral RNA synthesis and virus yield, an effect reversed by exogenous addition of guanosine. However, multiple mechanisms of action have been proposed for RIB according to the virus/host system analyzed [36,37]. In the particular case of the arenaviruses, it is currently in discussion which is the real mode of action for RIB. There is evidence for JUNV and LASV that the mechanism of action of RIB is not mainly focused on IMPDH inhibition and additional proposed targets may involve the viral RNA polymerase or lethal mutagenesis [38,39,40,41]. 

To overcome the shortcomings reported by the treatment of humans with RIB [9,10], other known IMPDH inhibitors were evaluated against different arenaviruses decades ago with variable results [42]. More recently, 5-ethynyl-1-β-D-ribofuranosylimidazole-4-carboxamide (EICAR) and mycophenolic acid (MPA) were successfully tested against pathogenic arenaviruses showing greater antiviral potency and selectivity than RIB *in vitro* against JUNV and LASV with MPA as the most active inhibitor (IC_50_ values around 0.3 µg/mL) [38,39]. EICAR is a competitive inhibitor of IMPDH, whereas MPA is an uncompetitive inhibitor, and both compounds possess antiproliferative, immunosuppressive and antiviral properties [43,44]. JUNV and LASV inhibition was highly reversed by exogenous guanosine addition, indicating that antiviral activity is effectively associated with GTP depletion through IMPDH blockade as well as the high susceptibility of both HF arenavirus replication to this cellular pathway of inhibition [38,39].

Acridones are another class of compounds apparently related to cellular targets that have attracted attention in recent years for their wide range of biological properties. As for the antiviral properties of this class of compounds, several studies have shown the inhibitory action of acridone derivatives against DNA viruses such as herpes simplex virus and cytomegalovirus [45,46], Epstein-Barr virus [47] and adenovirus [48], as well as RNA viruses, including human immunodeficiency virus [49,50], bovine viral diarrhea virus [51] and hepatitis C [52,53]. The precise target and the mode of action of antiviral acridones are not clearly determined at present, although its predominant action seems to be centered on the synthesis of nucleic acids. It has been identified specific inhibitory activity against cellular enzymes such as DNA topoisomerase II [45,54] and IMPDH [55], and viral enzymes such as RNA helicase and RNA polymerase [53] as well as the ability to intercalate into the nucleic acid molecules, blocking the recognition and association of the enzyme to the modified molecule [56]. 

With respect to arenaviruses, a screening of antiviral activity of diverse novel *N*-substituted acridone derivatives identified a group of 10-allyl-9(10*H*)-acridones as effective and very selective inhibitors of arenaviruses [57]. In particular, the 10-allyl-6-chloro-4-methoxy-9(10*H*)-acridone, designated **3f**, was the most active compound that blocked replication of the pathogenic arenaviruses JUNV and LCMV as well as the four serotypes of dengue virus, another HF-causing RNA virus belonging to the family *Flaviviridae*. Mechanistic studies demonstrated that JUNV RNA synthesis was strongly inhibited and the addition of exogenous guanosine rescued the infectivity and viral RNA levels in a dose-dependent manner [39]. However, the guanosine reversal was partial, suggesting that GTP pool reduction may contribute to the inhibitory action but it is not the only operative mechanism: a possible cellular target is represented by IMPDH whereas another still unidentified target, presumably viral, given the high selectivity of the compound, is also involved in the antiviral activity of the acridone against JUNV. Then, the possibility of a multiple effect on different targets, as reported for other acridones [53], turns promising perspectives for these agents in treatment of pathogenic arenavirus infections allowing a potent antiviral efficiency and less feasibility of generating resistance. 

Inhibitors of the biosynthetic pathway of pyrimidine nucleotides were also studied for anti-arenavirus activity. Compounds evaluated include cytidine analogs targeted to cytosine triphosphate synthetase (CTPS); carbanucleoside analogs which are inhibitors of the orotidylic acid decarboxylase (OMPD) which converts OMP to uridine monophosphate (UMP); and analogs of adenosine, most of them acting as inhibitors of S-adenosylhomocysteine hydrolase (SAHH), a key enzyme in the transmethylation to obtain the 5 ’cap of the mRNA [42,58,59]. However, the selectivity and effectiveness of these series of analogs were not very promising without showing appreciable advantages over RIB. 

The finding and characterization of molecules with broad-spectrum inhibitory action against viral RNA synthesis and minor cellular toxicity, as MPA and acridones above commented, represent novel promising alternatives for chemotherapy, although further *in vivo* evaluation is necessary to renew the consideration of this class of metabolic inhibitors against arenaviruses.

### 3.2. Heterogeneous Nuclear Ribonucleoproteins

Heterogeneous nuclear ribonucleoproteins (hnRNPs) constitute a family of cellular RNA binding proteins which participate in RNA metabolic processes including mRNA splicing, nucleo-cytoplasmic trafficking, translation and turnover [60,61,62]. More than 20 hnRNPs have been characterized, called from hnRNP A1 to hnRNP U, and those belonging to the A/B group (hnRNP A1, A2/B1, A3) are among the smallest but most abundant hnRNPs [62]. In virus-infected cells, several hnRNPs are able to interact with viral components, RNA and proteins, modulating the multiplication of both DNA and RNA viruses. Several studies have proved the involvement of hnRNPs in different stages of viral replicative cycle, mainly in RNA synthesis, processing and translation [63,64,65]. In addition, several viruses induce changes in the level of expression and intracellular localization of these predominantly nuclear proteins and these alterations may facilitate viral replication and/or help the virus to counteract the host cell antiviral response [66,67].

There is some evidence about changes in hnRNP expression in arenavirus-infected cells that suggest a possible role of these cell factors on both viral infectivity and/or pathogenesis. Pichinde virus (PICV) infection of guinea pigs produces a pathology similar to Lassa fever in humans [68] and two viral variants, P2 (an attenuated virus that causes a mild infection) and P18 (a virulent virus that produces a severe HF in the guinea pig model), have been used to analyze cell signaling pathways and responses following infection of a murine macrophage-like cell line [28,29,69]. These studies identified several cellular signaling events which may be involved in PICV pathogenesis [28,29]. Virus-host interactions were further characterized by investigating the differential nuclear proteomes induced by the infection of these two PICV variants [69]. This analysis revealed that several hnRNPs (hnRNP A2/B1, hnRNP A3, hnRNP D0 and hnRNP L) exhibited substantial differential expression after infection with the attenuated or the virulent isolates. The subcellular localization of hnRNPs during infection was also investigated: at early times after infection only the attenuated variant P2 induced the cytoplasmic localization of hnRNP A2/B1, whereas hnRNP A1 remained predominantly nuclear for both P2 and P18. The results obtained with hnRNP A2/B1 are in accordance with previous data showing that at the initial stages of the infection cells infected with the virulent P18 variant resemble uninfected cells. Bowick *et al.*, proposed either a failure in the activation of cell response or the suppression of this response after infection with the virulent virus, suggesting that virus-mediated alterations in hnRNP intracellular localization may be implicated in PICV pathogenesis [69]. 

Infection of macaques with a lethal dose of LCMV also constitutes a model for LASV infection of humans [70] and the analysis of the expression of cell genes in a blood sample by using DNA microarray technology made possible the identification of genes that may serve as markers for disease progression and to gain insights into the molecular mechanisms triggering viral HF. Moreover, the comparison of the blood profiles after infection with the virulent LCMV strain WE or the non-virulent strain Armstrong [71] enabled the finding of genes that would be associated with viral pathogenesis [72]. This comparative study revealed that hnRNP C is downregulated in peripheral blood mononuclear cells isolated at early times after infection from rhesus macaques that were infected with WE [72]. It is interesting to note that virus-mediated changes in hnRNP C expression or localization would result in the inhibition of interferon (IFN) signaling since this hnRNP has been proved to be involved in phospho-STAT1 nuclear import, so a reduced level of hnRNP C in WE-infected cells may contribute to the severity of the infection [63,67].

The first report providing evidence about the importance of hnRNPs on JUNV replication was a comparative study of acute and persistent JUNV infections performed in Vero cell cultures [73]. Silencing of hnRNP A1 or hnRNP A2, by using small interfering RNAs (siRNAs), caused a decrease in JUNV protein synthesis and progeny virus production during acute infection. It was also demonstrated that acute JUNV infection does not affect hnRNP A/B levels of expression in comparison with uninfected cells and promotes the cytoplasmic accumulation of hnRNPA1. Cytoplasmic re-distribution of hnRNP A1 could also be detected in cells expressing the viral nucleoprotein and co-immunoprecipitation studies revealed the interaction between both proteins. Unlike the acute infection, persistently infected cultures do not produce infectious virus and exhibit a continuous synthesis of nucleoprotein, associated with blockage of the expression of the glycoprotein G1 and absence of cytopathic effect [74,75]. A marked diminishment in the hnRNP A/B expression was observed in persistently JUNV-infected cultures. Furthermore, overexpression of hnRNP A1 allowed determining that cells persistently infected with JUNV do not induce hnRNP A1 cytoplasmic re-localization. These results indicate that hnRNP A1 would favor JUNV productive infection through the interaction with the nucleoprotein. On the other hand, JUNV non-productive persistent infection might involve downregulation of hnRNPs A/B, as well as changes in nucleo-cytoplasmic trafficking [73].

Despite much remains to be investigated about the relation between hnRNPs and arenavirus multiplication, persistence and pathogenesis, the data available to date indicate that these molecules can be considered as possible targets for antiviral therapy. However, it should be noted that hnRNPs do not always act as promoters of viral replication [63] and there are situations, such as hnRNP C downregulation by LCMV-WE infection mentioned above [72], in which a decrease in the level of expression of these cell factors could be associated with an increased viral pathogenicity. 

## 4. Host Kinases

### 4.1. Kinases and Signaling Pathways

The phosphorylation of proteins, either viral or cellular, is an event of key importance that determines the fate of several features during the infection process related to viral multiplication and cell survival as well. Phosphorylation is a reversible post-translational modification that proteins may suffer in order to regulate stability, biological activity and interactions with other proteins. This process is chemically characterized by the addition of a negatively charged phosphate group to specific serine or threonine residues of the protein and may be accomplished by cellular or viral kinases. On the contrary, removal of phosphate groups from protein backbone is achieved through the action of phosphatases which, together with kinases, control most of the cellular signaling pathways involved in metabolism, transcription and translation, cell cycle, apoptosis, differentiation and immune response [76,77].

Solid evidence shows that viruses require the modulation of cell signaling pathways in order to ensure their successful replication. In this context, modulation of cell survival pathways is a key target for both DNA and RNA viruses as a strategy to temporarily overcome cell death and prolonging viral replication. The phosphatidylinositol-3-kinase/protein kinase B (PI3K/Akt) is one of the major signaling pathways involved in cell survival, as well as proliferation, migration and apoptosis and, as such, has been shown to be widely involved in critical viral events like virus uptake, replication, translation and budding [78,79,80]. Several signaling pathways are also modulated by PI3K/Akt, among which are Bad, caspase-9, IKK, the Forkhead transcription factors, Mdm2, YAP, mTOR and the Forkhead box O (FoxO) transcription factors [81,82] and hence may be potentially regulated by viruses through PI3K modulation. 

It has been reported that JUNV is able to activate Akt-phosphorylation mediated by PI3K at an early stage of infection. This activation triggered during virus uptake would be necessary for an efficient recycling of transferrin receptor, main receptor involved in JUNV binding to the cell surface [83,84]. The inhibition of Akt phosphorylation by the PI3K inhibitor Ly294002 impaired virus production in different cell types indicating the essential role of PI3K/Akt signaling for JUNV infection. In view that PI3K/Akt activation is also involved in the stabilization of the mRNA encoding hnRNPA1 [85] and taking into account, as previously mentioned, the participation of this factor during JUNV replication [73], it is tempting to speculate that activation of this pathway may contribute in other stages of virus multiplication besides virus uptake. 

The participation of PI3K during the early stages of LASV and LCMV infection has also been described. For these Old World arenaviruses, which use α-dystroglycan as cellular receptor [86], the biosynthesis of lipid membrane phosphatidylinositol 3-phosphate (PI3P), mediated by PI3K, would be necessary to support the formation of multivesicular bodies, mechanism employed by these viruses to invade host cells [87]. In contrast, in a recent work it has been reported that inhibition of PI3K/Akt, but not of the downstream effector mTOR/raptor, during LCMV infection impaired budding and, at a lesser extent, RNA synthesis but not virus entry. Moreover, inhibition of PI3K/Akt reduced virus budding mediated by the matrix protein Z in a dose- dependent manner, suggesting the involvement of this cellular pathway in virus budding [88]. This apparent discrepancy about the involvement of PI3K in LCMV entry may be ascribed to differences in LCMV strains employed for each report as well as to the different mechanisms of action of PI3K inhibitors used by both groups. Noticeably, Urata *et al.*, demonstrated that BEZ-235, a PI3K inhibitor currently in cancer clinical trials, inhibited LCMV multiplication in cultured cells, reinforcing the good perspectives of cell-targeted agents in general and PI3K blockade in particular for arenavirus chemotherapy [88].

Interestingly, modulation of PI3K/Akt pathway was observed in CD8+ T cells during acute LCMV infection. *In vivo* phosphorylation levels of Akt, mTOR and FoxO1/O3, a critical regulator of T cell homeostasis activated by PI3K/Akt pathway, showed dynamic alterations during the course of infection: while Akt activation correlated with mTOR activity, no correlation was observed in FoxO1/O3 phosphorylation, inhibiting thus apoptosis of infected lymphocytes [89]. 

As mentioned above, the comparison of signaling proteins regulated during macrophage infection with attenuated or virulent strains of PICV also allowed detect a differential response to each strain in three of the central signaling networks involving p53, c-Myc, and Akt, respectively [28]. Further studies revealed that macrophages isolated from guinea pigs infected with the P2 or P18 strain of PICV showed differential phosphorylation status of ERK [29], a component of the RAF-MEK-ERK1/2 signaling pathway, other central route that regulates many cellular functions related to virus multiplication [90]. Although this pathway controls cell differentiation and proliferation leading to changes in gene expression, cell metabolism and apoptosis, little is known about its modulation by arenaviruses. In this regard, increase in ERK phosphorylation has been reported during PICV multiplication [91]. On the contrary, LASV infection impaired MEK/ERK activation after virus binding to its receptor [92]. 

The trafficking mechanism of arenavirus is also an important point to be considered as a possible cellular target [93]. Upon binding receptor, endosomal acidification is a mandatory step for internalization of either New or Old world arenaviruses [94,95,96,97], although cellular receptor and hence the trafficking mechanisms are different. In case of LCMV and LASV, virus internalization is cholesterol-dependent but is independent of clathrin, caveolin, and dynamin. On the other hand the proteins Rab-5 and Rab-7 are partially required while ARF6, flotillin and actin would not to be necessary for LCMV and LASV entry [98,99,100,101]. On the contrary for the New World arenaviruses JUNV and PICV, virus internalization is clathrin- and dynamin-dependent but is partially dependent of cholesterol and independent of caveola. The proteins Eps15, Rab-5 and Rab-7 have also been shown necessary for both viruses [102,103,104]. In this regard, although novel approaches have been evaluated for antiviral drug development [105], the possibility to consider inhibition of kinases participating in the internalization process remains in the speculative field [106].

In conclusion, the modulation of survival signaling pathways is a critical event in the replication cycle of arenavirus. Given that these pathways are highly involved in the cell deregulation that occurs during cancer, much progress has been obtained in the development of potential chemotherapy agents against different components of these pathways that might be used as anti-arenaviral drugs. 

### 4.2. Kinases and Interferon

The host immune response has been widely accepted to be one of the essential factors to determine the outcome of LCMV infection in mice [107]. From many of these studies it can be concluded that viral modulation of phosphorylation of proteins involved in the IFN response is a key event to explain pathogenicity. 

Type I interferon (IFN-I) has been pointed out as an important factor that may have beneficial or detrimental consequences during LCMV infection of mouse [108]. On this basis, it has been demonstrated that inhibition of the innate immune response, mainly supported by the production of IFN, is necessary for the establishment of viral persistence in the mouse [109]. *In vitro* infection of several cell types by different arenaviruses showed the induction of IFN-I [110,111]. It has been reported that LCMV nucleoprotein negatively modulates IFN production in persistently infected A549 cells by inhibiting nuclear translocation of the interferon regulatory factor 3 (IRF-3) [112]. This property has been also demonstrated for nucleoproteins of several representatives of New and Old World arenaviruses except for Tacaribe virus nucleoprotein [113]. LCMV nucleoprotein is able to interact with the IκB kinase (IKK)-related kinase IKKε leading to a loss of its phosphorylation activity towards IRF-3 [114]. At the same time, LCMV nucleoprotein is able to interfere with the nuclear translocation of the nuclear factor kappa B (NF-κ B) suggesting a multi-factorial role of this protein in the modulation of innate immune response and inflammation [115]. Also, Z protein from New World arenaviruses, but not from Old World members, antagonize IFN response by binding to the retinoic acid-inducible gene I (RIG-I) and inhibiting downstream activation of IFN-I expression signaling pathway [116].

One of the main mechanisms mediated by IFN and employed by cells to restrict viral infection is the up-regulation of the protein kinase R (PKR), which is activated by double-stranded RNA, a species that can be readily detected during the replication of many viruses. PKR expression is augmented during the IFN response and, in the case of infection, it phosphorylates the α subunit of eukaryotic translation initiation factor 2 (eIF2α), resulting in the blockage of cap dependent translation and impairment of the bulk of cellular protein synthesis and in many cases, viral protein synthesis as well [117]. At the same time, phosphorylation of eIF2α leads to the formation of stress granules, cytoplasmic aggregates that contain pre-initiation complexes that accumulate as a consequence of translation blockage. Many viruses can modulate eIF2α phosphorylation in order to counteract cell restriction [118]. On this line, it has been reported that JUNV is able to impair eIF2α phosphorylation upon stressing of infected Vero cells with sodium arsenite. Both, the nucleoprotein and the glycoprotein precursor were able to maintain low eIF2α phosphorylation levels, similar to those observed for control cells [119]. Although the mechanism through which arsenite induces eIF2α phosphorylation is under discussion, PKR may be one of the kinase targets for this drug [120].

Another point that might be considered as a prospective target for anti-arenaviral therapy is the Toll-like receptor 2 (TLR2) pathway that activates host defense mechanisms in order to prevent and eradicate infections and participates in the generation of the adaptive immune response [121]. Several years ago, Zhou *et al.*, studied the role of TLR2 and myeloid differentiation factor 88 (MyD88) during an LCMV infection and demonstrated that TLR-mediated responses are necessary for an efficient innate immune response against this virus and that MyD88 would be of crucial importance for the maturation and activation of virus-specific CD8 (+) T cells [122]. Further studies revealed that LCMV infection of glial cells leads to the production of inflammatory chemokines, such as MCP-1, RANTES and TNF-alpha in a TLR2-MyD88 dependent manner [123]. On this perspective, a comprehensive study of prospective inhibitors of TLR2 signaling described the characterization of one of the active compounds that also exhibited antiviral properties against LCMV, suggesting that this approach may be a promising tool for anti-arenaviral therapy [124]. It should be emphasized that the studies mentioned above were performed with the Armstrong strain of LCMV. Recent findings demonstrate that the WE strain of LCMV and LASV, both able to induce a fatal disease in rhesus macaques, suppressed pro-inflammatory cytokine responses that depend on TLR2. By contrast, nonpathogenic Mopeia arenavirus is able to activate TLR2 pathway, as described for the Armstrong strain of LCMV [125]. Similar results were reported for JUNV infection of murine macrophages, where expression of the TNF-alpha and beta IFN through a TLR2 pathway are activated by the viral glycoproteins [110].

In view of the results obtained to the present, impairment/disruption of innate immune response would be a key parameter that controls arenavirus pathogenicity. This approach deserves further investigation in order to determine if kinases involved in the modulation of IFN production/response would be attractive targets for antiviral therapy.

## 5. Intrinsic Antiviral Resistance

Intrinsic antiviral resistance, also known as viral restriction factors or intrinsic immunity, is a more recently described antiviral defense. Unlike acquired immunity and cytokine mediated innate immunity, intrinsic resistance functions through constitutively expressed cellular proteins that restrict virus replication within an individual infected cell [126,127]. Intrinsic resistance involves diverse proteins and mechanisms, depending on the particular virus. This group of proteins with potent antiviral properties is also induced by IFN-I [128] and comprised unrelated proteins like viperin and tetherin, among others [129,130,131]. 

In this context, it has been reported that components of nuclear structures known as promyelocytic leukemia protein nuclear bodies (PML NBs) contribute to intrinsic resistance against a variety of DNA and RNA viruses, including arenaviruses [132]. PML NBs are macromolecular protein assemblies implicated in key cellular functions including cell cycle progression, DNA damage response, transcriptional regulation and apoptosis, but the precise biochemical function of PML NBs in these processes is not known [133,134]. PML NBs range in size from 0.2 to 1.2 μm in diameter [135] and their number and distribution vary considerably depending on cell type, cell cycle and cell condition, but typically between 10 and 20 PML NBs can be found per nucleus [136]. 

Interestingly, PML NBs have been shown to contribute to innate defense against a broad range of viruses, and it has been reported that IFN treatment increases the number and size of PML NBs [137,138,139]. In turn, many viruses encode products that modify or eliminate PML NBs in cultured cells [132]. In particular for arenaviruses, it has been reported that LCMV influences the distribution of PML NBs through the specific interaction of the arenaviral Z protein and PML to form large bodies primarily in the cytoplasm of infected cells. Transient transfection and co-immunoprecipitation studies indicated that Z alone is sufficient to redistribute PML to the cytoplasm [140], whereas Z protein unfolded and inactivated by the effect of a zinc finger reactive compound failed to modify the distribution pattern of PML NBs components [141]. Moreover, the pro-apoptotic activity of PML in LCMV infected cells is mediated through the PML zinc binding region and the viral attack of PML NBs would allow the virus to deregulate host cell apoptotic machinery in order to establish chronic infection [142]. 

More importantly, another report has described that LCMV multiplication in PML −/− murine embryonic fibroblasts (MEF) exceeds virus production in PML +/+ MEF [116], and that *in vivo* PML deficiency renders mice more susceptible to different viral infections, such as vesicular stomatitis virus, encephalomyocarditis virus and LCMV infection, resulting in an increased viral replication [144,145].

**Figure 2:**
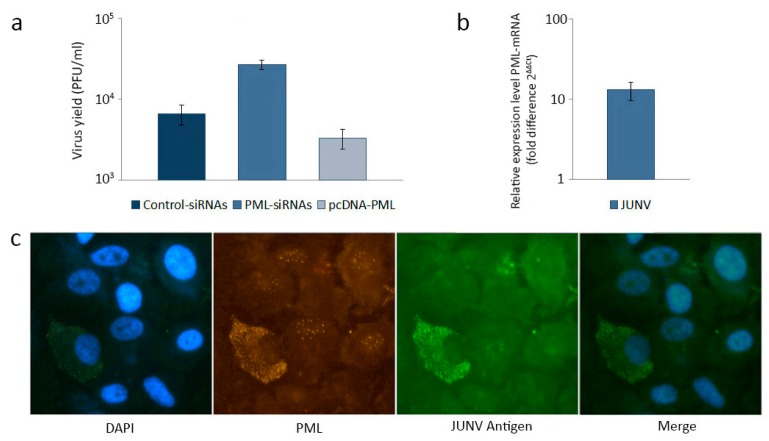
(**a**) Effect of PML on infectious particle production. Control-siRNAs, PML-siRNAs and pcDNA-PML transfected A549 cells were infected with an attenuated strain of JUNV. At 24 h post infection (p.i.) viral yields were determined by a standard plaque assay; (**b**) Determination of PML-mRNA expression levels. JUNV infected A549 cells were harvested at 24 h p.i. for qRT-PCR. PML-mRNA expression was represented as fold difference relative to mock infected cells and normalized to β-actin-mRNA; (**c**) Effect of JUNV infection on PML-NBs distribution. JUNV infected A549 cells were fixed 24 h p.i. and a double immunofluorescence was performed using monoclonal anti-nucleoprotein antibody visualized by fluorescein isothiocyanate and monoclonal anti-PML antibody visualized by tetramethyl rhodamine isothiocyanate. Cell nuclei were stained with 4',6-diamidino-2-phenylindole (DAPI) (400X).

In order to expand these studies, the antiviral role of PML NBs in JUNV multiplication was explored by determining the impact of PML silencing with specific siRNAs on infectious particle production. The virus yield observed in PML-silenced A549 cells was 4-fold higher than virus yield obtained in A549 control-siRNAs transfected cells (Figure 2a). The effective silencing of PML was confirmed by qRT-PCR (not shown), and, as seen, rendered these cells more susceptible to JUNV infection. To complement these findings, A549 cells were transfected with a PML encoding plasmid (pcDNA-PML), and then infected with JUNV. It was observed that PML overexpression induced partial resistance to JUNV multiplication, as 50% inhibition on virus yield was obtained (Figure 2a). The restrictive role of PML in JUNV infection was also inferred through the determination of PML-mRNA by qRT-PCR in uninfected and JUNV infected cells. As seen in Figure 2b, JUNV infection produced more than 10-fold increase in PML-mRNA expression relative to control A549 cells, confirming the contribution of PML to cellular antiviral defense against JUNV multiplication. Finally, confocal images of a A549 cell culture infected with JUNV illustrate the differential PML pattern between viral antigen-negative and viral antigen-positive cells (Figure 2c). Cells negative for JUNV antigen expression showed the typical punctuate pattern of PML NBs. In contrast, in cells expressing JUNV antigen a dramatic disruption of PML NBs was observed, with a marked efflux of PML protein from the nucleus to the cytoplasm, remaining only a diffuse fluorescence in the vicinity of the original nuclear structure. 

Altogether these results are the first evidence of PML contribution to a cellular antiviral response against an attenuated strain of JUNV; however, the mechanism by which PML inhibits this arenavirus still is unknown, and it is not clear that these effects observed *in vitro* could impact in viral disease. To further determine the biological impact of PML on JUNV replication, it will be necessary to look at the ability of arenaviruses to persist or cause disease in PML-knock down animals. 

It seems that the constitutive expression of PML would allow for immediate antiviral activity of this molecule, an effect which could be potentiated upon upregulation through IFN, or indirectly participate in the IFN-mediated upregulation of known or yet unknown IFN-regulated proteins with antiviral activity. Future studies with extensive and detailed molecular work will be needed to dissect this large array of potentially redundant and parallel pathways. 

## 6. Conclusions and Future Perspectives

The alternative of cellular targets for antiviral chemotherapy against arenaviruses is at the initial phase of development, intending to identify the more appropriate virus-host cell interactions for an accurate blockade of infection regarding effectiveness and selectivity. As it was commented here, several host factors and also molecules directed to them have just been characterized *in vitro* and represent a promising starting point for future development. The innovative technologies of reverse genetics suitable for drug targeting by HTS, recently developed for arenaviruses, in combination with the analysis of transcriptome profiles of infected cells open new avenues in this field and may lead in the near future to an accelerated identification of novel key host factors in regulatory networks important for virus propagation. However challenges remain to be investigated in the next years about the real biological impact of this cell-based strategy for prophylaxis and treatment of the human disease, by the crucial testing of these indirect antiviral agents in adequate animal models. Those candidate drugs in clinical use or in an advanced stage of development for other diseases present the additional advantage of the knowledge of their toxicological and pharmacological profile. But also the novel molecules targeted to cellular pathways still not evaluated *in vivo* and found effective inhibitors *in vitro* assays represent an attractive starting point for *in vivo* testing, given the perspective of a broad-spectrum activity against HF arenaviruses in continued and growing emergency. 

## References

[1] McCormick J.B., Fisher-Hoch S.P. (2002). Lassa fever. Curr. Top. Microbiol. Immunol..

[2] Enria D.A., Briggiler A.M., Sánchez Z. (2008). Treatment of Argentine hemorrhagic fever. Antivir. Res..

[3] Macher A.M., Wolfe M.S. (2006). Historical Lassa fever reports and 30-year clinical update. Emerg. Infect. Dis..

[4] Jamieson D.J., Kourtis A.P., Bell M., Rasmussen S.A. (2006). Lymphocytic choriomeningitis virus: An emerging obstetric pathogen?. Am. J. Obstet. Gynecol..

[5] Fisher S.A., Graham M.B., Kuehnert M.J., Kotton C.N., Srinivasan A., Marty F.M., Comer J.A., Guarner J., Paddock C.D., DeMeo D.L. (2006). LCMV in Transplant recipients investigation team. Transmission of lymphocytic choriomeningiris virus by organ transplantation. N. Engl. J. Med..

[6] Charrel R.N., de Lamballerie X. (2010). Zoonotic aspects of arenavirus infections. Vet. Microbiol..

[7] McCormick J.B., King I.J., Webb P.A., Scribner C.L., Craven R.B., Johnson K.M., Elliot L.H., Belmont-Williams R. (1986). Lassa fever. Effective therapy with ribavirin. N. Engl. J. Med..

[8] Peters C.J. (2002). Human infection with arenaviruses in the Americas. Curr. Top. Microbiol. Immunol..

[9] Fisher-Hoch S.P., Ghorie S., Parker L., Huggins J. (1992). Unexpected adverse reactions during a clinical trial in rural West Africa. Antivir. Res..

[10] Enria D.A., Maiztegui J.I. (1994). Antiviral treatment of Argentine hemorrhagic fever. Antivir. Res..

[11] Duffy S., Shackelton L.A., Holmes E.C. (2008). Rates of evolutionary change in viruses: Patterns and determinants. Nat. Rev. Genet..

[12] Sanjuán R., Nebot M.R., Chirico N., Mansky L.M., Belshaw R. (2010). Viral mutation rates. J. Virol..

[13] Coley W., Kehn-Hall K., van Duyne R., Kashanchi F. (2009). Novel HIV-1 therapeutics through targeting altered host cell pathways. Expert Opin. Biol. Ther..

[14] Khattab M.A. (2009). Targeting host factors: A novel rationale for the management of hepatitis C virus. World J. Gastroenterol..

[15] Krumm S.A., Ndungu J.M., Yoon J.J., Dochow M., Sun A., Natchus M., Snyder J.P., Plemper R.K. (2011). Potent host-directed small-molecule inhibitors of myxovirus RNA-dependent RNA-polymerases. PLoS One.

[16] Pastorino B., Nougairede A., Wurtz N., Gould E., de Lamballerie X. (2010). Role of host cell factors in flavivirus infection: Implications for pathogenesis and development of antiviral drugs. Antivir. Res..

[17] Albariño C.G., Bergeron E., Erickson B.R., Khristova M.L., Rollin P.E., Nichol S.T. (2009). Efficient reverse genetics generation of infectious junin viruses differing in glycoprotein processing. J. Virol..

[18] Emonet S.F., Seregin A.V., Yun N.E., Poussard A.L., Walker A.G., de la Torre J.C., Paessler S. (2011). Rescue from cloned cDNAs and *in vivo* characterization of recombinant pathogenic Romero and live-attenuated Candid #1 strains of Junin virus, the causative agent of Argentine hemorrhagic fever disease. J. Virol..

[19] Carnec X., Baize S., Reynard S., Diancourt L., Caro V., Tordo N., Bouloy M. (2011). Lassa virus nucleoprotein mutants generated by reverse genetics induce a robust type I interferon response in human dendritic cells and macrophages. J. Virol..

[20] Larson R.A., Dai D., Hosack V.T., Tan Y., Bolken T.C., Hruby D.E., Amberg S.M. (2008). Identification of a broad-spectrum arenavirus entry inhibitor. J. Virol..

[21] Lee A.M., Rojek J.M., Spiropoulou C.F., Gundersen A.T., Jin W., Shaginian A., York J., Nunberg J.H., Boger D.L., Oldstone M.B. (2008). Unique small molecule entry inhibitors of hemorrhagic fever arenaviruses. J. Biol. Chem..

[22] Capul A.A., de la Torre J.C. (2008). A cell-based luciferase assay amenable to high-throughput screening of arenavirus budding. Virology.

[23] Friedel C.C., Jurgen H. (2011). Virus-Host interactomes and global models of virus-infected cells. Trends Microbiol..

[24] Shaw M.L. (2011). The host interactome of influenza virus presents new potential targets for antiviral drugs. Res. Med. Virol..

[25] Tafforeau L., Rabourdin-Combe C., Lotteau V. (2012). Virus-Human cell interactomes. Methods Mol. Biol..

[26] Djavani M., Crasta O.R., Zhang Y., Zapata J.C., Sobral B., Lechner M.G., Bryant J., Davis H., Salvato M.S. (2009). Gene expression in primate liver during viral hemorrhagic fever. Virol. J..

[27] Müller S., Geffers R., Günther S. (2007). Analysis of gene expression in Lassa virus-infected HuH-7 cells. J. Gen. Virol..

[28] Bowick G.C., Fennewald S.M., Elsom B.L., Aronson J.F., Luxon B.A., Gorenstein D.G., Herzog N.K. (2006). Differential signaling networks induced by mild and lethal hemorrhagic fever virus infections. J. Virol..

[29] Bowick G.C., Fennewald S.M., Scott E.P., Zhang L., Elsom B.L., Aronson J.F., Spratt H.M., Luxon B.A., Gorenstein D.G., Herzog N.K. (2007). Identification of differentially activated cell-signaling networks associated with Pichinde virus pathogenesis by using systems kinomics. J. Virol..

[30] García C.C., Sepúlveda C.S., Damonte E.B. (2011). Novel therapeutic targets for arenavirus hemorrhagic fevers. Future Virol..

[31] Emonet S.E., Urata S., de la Torre J.C. (2011). Arenavirus reverse genetics: New approaches for the investigation of arenavirus biology and development of antiviral strategies. Virology.

[32] Lee A.M., Pasquato A., Kunz S. (2011). Novel approaches in anti-arenaviral drug development. Virology.

[33] Rojek J.M., Kunz S. (2008). Cell entry by human pathogenic arenaviruses. Cell. Microbiol..

[34] Streeter D.G., Witkowski J.T., Khare G.P., Sidwell R.W., Bauer R.J., Robins R.K., Simon L.N. (1973). Mechanism of action of 1-ß-D-ribofuranosyl-1,2,4-triazole-3- carboxamide (Virazole), a new broad-spectrum antiviral agent. Proc. Natl. Acad. Sci. USA.

[35] Leyssen P., Balzarini J., de Clercq E., Neyts J. (2005). The predominant mechanism by which ribavirin exerts its antiviral activity *in vitro* against flaviviruses and paramyxoviruses is mediated by inhibition of IMP dehydrogenase. J. Virol..

[36] Graci J.D., Cameron C.E. (2006). Mechanisms of action of ribavirin against distinct viruses. Rev. Med. Virol..

[37] Leyssen P., de Clercq E., Neyts J. (2008). Molecular strategies to inhibit the replication of RNA viruses. Antivir. Res..

[38] Ölschläger S., Neyts J., Günther S. (2011). Depletion of GTP pool is not the predominant mechanism by which ribavirin exerts its antiviral effect on Lassa virus. Antivir. Res..

[39] Sepúlveda C.S., García C.C., Fascio M.L., D’Accorso N.B., Docampo Palacios M.L., Pellón R F., Damonte E.B. (2012). Inhibition of Junín virus RNA synthesis by an antiviral acridone derivative. Antivir. Res..

[40] Ruiz-Jarabo C.M., Ly C., Domingo E., de la Torre J.C. (2003). Lethal mutagenesis of the prototype arenavirus lymphocytic choriomeningitis virus (LCMV). Virology.

[41] Moreno H., Gallego I., Sevilla N., de la Torre J.C., Domingo E., Martín V. (2011). Ribavirin can be mutagenic for arenaviruses. J. Virol..

[42] Andrei G., de Clercq E. (1993). Molecular approaches for the treatment of hemorrhagic fever virus infections. Antivir. Res..

[43] Minakawa N., Takeda T., Sasaki T., Matsuda A., Ueda T. (1991). Nucleotides and nucleosides. 96. Synthesis and antitumor activity of 5-ethynyl-1-β-D-ribofuranosylimidazole-4-carboxamide (EICAR) and its derivatives. J. Med. Chem..

[44] Sintchak M.D., Nimmesgern E. (2000). The structure of inosine 5X-monophosphate dehydrogenase and the design of novel inhibitors. Inmunopharmacology.

[45] Goodell J.R., Madhok A.A., Hiasa H., Ferguson D.M. (2006). Synthesis and evaluation of acridone- and acridone-based anti-herpes agents with topoisomerase activity. Bioorg. Med. Chem..

[46] Lowden C.T., Bastow K.F. (2003). Cell culture replication of herpes simplex virus and, or human cytomegalovirus is inhibited by 3,7-dialkoxylated, 1-hydroxyacridone derivatives. Antivir. Res..

[47] Itoigawa M., Ito C., Wu T.S., Enjo F., Tokuda H., Nishino H., Furukawa H. (2003). Cancer chemopreventive activity of acridone alkaloids on Epstein-Barr virus activation and two-stage mouse skin carcinogenesis. Cancer Lett..

[48] Zarubaev V.V., Slita A.V., Krivitskaya V.Z., Sirotkin A.K., Kovalenko A.L., Chatterjee N.K. (2003). Direct antiviral effect of cycloferon (10-carboxymethyl-9-acridanone) against adenovirus type 6 *in vitro*. Antivir. Res..

[49] Fujiwara M., Okamoto M., Okamoto M., Watanabe M., Machida H., Shigeta S., Konno K., Yokota T., Baba M. (1999). Acridone derivatives are selective inhibitors of HIV-1 replication in chronically infected cells. Antivir. Res..

[50] Turpin J.A., Buckheit R.W., Derse D., Hollingshead M., Williamson K., Palamone C., Osterling M.C., Hill S.A., Graham L., Schaeffer C.A. (1998). Inhibition of acute-, latent-, and chronic-phase human immunodeficiency virus type 1 (HIV-1) replication by a bistriazoloacridone analog that selectively inhibits HIV-1 transcription. Antimicrob. Agents Chemother..

[51] Tabarrini O., Manfroni G., Fravolini A., Cecchetti V., Sabatini S., de Clercq E., Rozenski J., Canard B., Dutartre H., Paeshuyse J. (2006). Synthesis and anti-BVDV activity of acridones as new potential antiviral agents. J. Med. Chem..

[52] Manfroni G., Paeshuyse J., Massari S., Zanoli S., Gatto B., Maga G., Tabarrini O., Cecchetti V., Fravolini A., Neyts J. (2009). Inhibition of subgenomic hepatitis C virus RNA replication by acridone derivatives: Identification of an NS3 helicase inhibitor. J. Med. Chem..

[53] Stankiewicz-Drogon A., Dörner B., Erker T., Boguszewska-Chachulska A.M. (2010). Synthesis of new acridone derivatives, inhibitors of NS3 helicase, which efficiently and specifically inhibit subgenomic HCV replication. J. Med. Chem..

[54] Vispé S., Vandenberghe I., Robin M., Annereau J.P., Créancier L., Pique V., Galy J.P., Kruczynski A., Barret J.M., Bailly C. (2007). Novel tetra-acridine derivatives as dual inhibitors of topoisomerase II and the human proteasome. Biochem. Pharmacol..

[55] Watterson S.H., Chen P., Zhao Y., Gu H.H., Dhar T.G., Xiao Z., Ballentine S.K., Shen Z., Fleener C.A., Rouleau K.A. (2007). Acridone-Based inhibitors of inosine 5’-monophosphate dehydrogenase: Discovery and SAR leading to the identification of N-(2-(6-(4-ethylpiperazin-1-yl)pyridin-3-yl)propan-2-yl)-2-fluoro-9-oxo-9,10-dihydroxyacridine-3-carboxamide (BMS-566419). J. Med. Chem..

[56] Adams A. (2002). Crystal structures of acridines complexed with nucleic acids. Curr. Med. Chem..

[57] Sepúlveda C.S., Fascio M.L., Mazzucco M.B., Palacios M.L., Pellón R.F., García C.C., D’Accorso N.B., Damonte E.B. (2008). Synthesis and evaluation of N-substituted acridones as antiviral agents against hemorrhagic fever viruses. Antivir. Chem. Chemother..

[58] Gowen B.B., Wong M.H., Larson D., Ye W., Jung K.H., Sefing E.J., Skirpstunas R., Smee D.F., Morrey J.D., Schneller S.W. (2010). Development of a new Tacaribe arenavirus infection model and its use to explore antiviral activity of a novel aristeromycin analog. PLoS One.

[59] Guillerm G., Guillerm D., Vandenplas-Vitkowski C., Glapski C., de Clercq E. (2003). Inactivation of S-adenosyl-L-homocysteine hydrolase with novel 5’-thioadenosine derivatives. Antiviral effects. Bioorg. Med. Chem. Lett..

[60] Venables J.P., Koh C.S., Froehlich U., Lapointe E., Couture S., Inkel L., Bramard A., Paquet E.R., Watier V., Durand M. (2008). Multiple and specific mRNA processing targets for the major human hnRNP proteins. Mol. Cell Biol..

[61] He Y., Smith R. (2009). Nuclear functions of heterogeneous nuclear ribonucleoproteins A/B. Cell. Mol. Life Sci..

[62] Han S.P., Tang Y.H., Smith R. (2010). Functional diversity of the hnRNPs: Past, present and perspectives. Biochem. J..

[63] Castilla V., Scolaro L.A. (2012). Involvement of heterogeneous nuclear ribonucleoproteins in viral multiplication. Future Virol..

[64] Katoh H., Mori Y., Kambara H., Abe T., Fukuhara T., Morita E., Moriishi K., Kamitani W., Matsuura Y. (2011). Heterogeneous nuclear ribonucleoprotein A2 participates in the replication of Japanese encephalitis virus through an interaction with viral proteins and RNA. J. Virol..

[65] Shih S.R., Stollar V., Li M.L. (2011). Host factors in enterovirus 71 replication. J. Virol..

[66] Monette A., Ajamian L., López-Lastra M., Mouland A.J. (2009). Human immunodeficiency virus type 1 (HIV-1) induces the cytoplasmic retention of heterogeneous nuclear ribonucleoprotein A1 by disrupting nuclear import: Implications for HIV-1 gene expression. J. Biol. Chem..

[67] Shabman R.S., Gulcicek E.E., Stone K.L., Basler C.F. (2011). The Ebola virus VP24 protein prevents hnRNP C1/C2 binding to karyopherin α1 and partially alters its nuclear import. J. Infect. Dis..

[68] Jahrling P.B., Hesse R.A., Rhoderick J.B., Elwell M.A., Moe J.B. (1981). Pathogenesis of a Pichinde virus strain adapted to produce lethal infections in guinea pigs. Infect. Immun..

[69] Bowick G.C., Spratt H.M., Hogg A.E., Endsley J.J., Wiktorowicz J.E., Kurosky A., Luxon B.A., Gorenstein D.G., Herzog N.K. (2009). Analysis of the differential host cell nuclear proteome induced by attenuated and virulent hemorrhagic arenavirus infection. J. Virol..

[70] Lukashevich I.S., Djavani M., Rodas J.D., Zapata J.C., Usborne A., Emerson C., Mitchen J., Jahrling P.B., Salvato M.S. (2002). Hemorrhagic fever occurs after intravenous, but not after intragastric, inoculation of rhesus macaques with lymphocytic choriomeningitis virus. J. Med. Virol..

[71] Lukashevich I.S., Rodas J.D., Tikhonov I.I., Zapata J.C., Yang Y., Djavani M., Salvato M.S. (2004). LCMV-mediated hepatitis in rhesus macaques: WE but not ARM strain activates hepatocytes and induces liver regeneration. Arch. Virol..

[72] Djavani M.M., Crasta O.R., Zapata J.C., Fei Z., Folkerts O., Sobral B., Swindells M., Bryant J. (2007). Early blood profiles of virus infection in a monkey model for Lassa fever. J. Virol..

[73] Maeto C.A., Knott M.E., Linero F.N., Ellenberg P.C., Scolaro L.A., Castilla V. (2011). Differential effect of acute and persistent Junin virus infections on the nucleo-cytoplasmic trafficking and expression of heterogeneous nuclear ribonucleoproteins type A and B. J. Gen. Virol..

[74] Ellenberg P., Edreira M., Lozano M., Scolaro L. (2002). Synthesis and expression of viral antigens in Vero cells persistently infected with Junin virus. Arch. Virol..

[75] Ellenberg P., Edreira M., Scolaro L. (2004). Resistance to superinfection of Vero cells persistently infected with Junin virus. Arch. Virol..

[76] Keating J.A., Striker R. (2012). Phosphorylation events during viral infections provide potential therapeutic targets. Rev. Med. Virol..

[77] Dissmeyer N., Schnittger A. (2011). The age of protein kinases. Methods Mol. Biol..

[78] Buchkovich N.J., Yu Y., Zampieri C.A., Alwine J.C. (2008). The TORrid affairs of viruses: Effects of mammalian DNA viruses on the PI3K-Akt-mTOR signalling pathway. Nat. Rev. Microbiol..

[79] Cooray S. (2004). The pivotal role of phosphatidylinositol 3-kinase-Akt signal transduction in virus survival. J. Gen. Virol..

[80] Ji W.T., Liu H.J. (2008). PI3K-Akt signaling and viral infection. Recent. Pat. Biotechnol..

[81] McCormick F. (2004). Cancer: Survival pathways meet their end. Nature.

[82] Zhang X., Tang N., Hadden T.J., Rishi A.K. (2011). Akt, FoxO and regulation of apoptosis. Biochim. Biophys. Acta.

[83] Linero F.N., Scolaro L.A. (2009). Participation of the phosphatidylinositol 3-kinase/Akt pathway in Junín virus replication *in vitro*. Virus Res..

[84] Radoshitzky S.R., Abraham J., Spiropoulou C.F., Kuhn J.H., Nguyen D., Li W., Nagel J., Schmidt P.J., Nunberg J.H., Andrews N.C. (2007). Transferrin receptor 1 is a cellular receptor for New World hemorrhagic fever arenaviruses. Nature.

[85] Ruggiero T., Trabucchi M., Ponassi M., Corte G., Chen C.Y., al-Haj L., Khabar K.S., Briata P., Gherzi R. (2007). Identification of a set of KSRP target transcripts upregulated by PI3K-AKT signaling. BMC Mol. Biol..

[86] Cao W., Henry M.D., Borrow P., Yamada H., Elder J.H., Ravkov E.V., Nichol S.T., Compans R.W., Campbell K.P., Oldstone M.B. (1998). Identification of alpha-dystroglycan as a receptor for lymphocytic choriomeningitis virus and Lassa fever virus. Science.

[87] Pasqual G., Rojek J.M., Masin M., Chatton J.Y., Kunz S. (2011). Old world arenaviruses enter the host cell via the multivesicular body and depend on the endosomal sorting complex required for transport. PLoS Pathog..

[88] Urata S., Ngo N., de la Torre J.C. (2012). The PI3K/Akt pathway contributes to arenavirus budding. J. Virol..

[89] Sullivan J.A., Kim E.H., Plisch E.H., Peng S.L., Suresh M. (2012). FOXO3 regulates CD8 T cell memory by T cell-intrinsic mechanisms. PLoS Pathog..

[90] Pleschka S. (2008). RNA viruses and the mitogenic Raf/MEK/ERK signal transduction cascade. Biol. Chem..

[91] Vela E.M., Bowick G.C., Herzog N.K., Aronson J.F. (2008). Genistein treatment of cells inhibits arenavirus infection. Antivir. Res..

[92] Rojek J.M., Moraz M.L., Pythoud C., Rothenberger S., van der Goot F.G., Campbell K.P., Kunz S. (2012). Binding of Lassa virus perturbs extracellular matrix-induced signal transduction via dystroglycan. Cell. Microbiol..

[93] Vela E.M., Bowick G.C., Herzog N.K., Aronson J.F. (2008). Exploring kinase inhibitors as therapies for human arenavirus Infections. Future Virol..

[94] Castilla V., Merisch S. (1996). Low pH-induced fusion of Vero cells infected with Junin virus. Arch. Virol..

[95] Di Simone C., Zandonatti M., Buchmeier M. (1994). Acidic pH triggers LCMV membrane fusion activity and conformational change in the glycoprotein spike. Virology.

[96] Di Simone C., Buchmeier M. (1995). Kinetics and pH dependence of acid-induced structural changes in the Lymphoytic choriomeningitis virus glycoprotein complex. Virology.

[97] Borrow P., Oldstone M.B. (1994). Mechanism of lymphocytic choriomeningitis virus entry into cells. Virology.

[98] Shah W.A., Peng H., Carbonetto S. (2006). Role of non-raft cholesterol in lymphocytic choriomeningitis virus infection via {alpha}-dystroglycan. J. Gen. Virol..

[99] Quirin K., Eschli B., Scheu I., Poort L., Kartenbeck J., Helenius A. (2008). Lymphocytic choriomeningitis virus uses a novel endocytic pathway for infectious entry via late endosomes. Virology.

[100] Rojek J.M., Perez M., Kunz S. (2008). Cellular entry of lymphocytic choriomeningitis virus. J. Virol..

[101] Rojek J.M., Sanchez A.B., Nguyen N.T., de la Torre J.C., Kunz S. (2008). Different mechanisms of cell entry by human-pathogenic Old World and New World arenaviruses. J. Virol..

[102] Martinez M.G., Cordo S.M., Candurra N.A. (2007). Characterization of JUNV arenavirus cell entry. J. Gen. Virol..

[103] Vela E.M., Colpitts T.M., Zhang L., Davey R.A., Aronson J.F. (2008). Pichinde virus is trafficked through a dynamin 2 endocytic pathway that is dependent on cellular Rab5- and Rab7-mediated endosomes. Arch. Virol..

[104] Martínez M.G., Forlenza M.B., Candurra N.A. (2009). Involvement of cellular proteins in Junin arenavirus entry. Martinez MG, Forlenza MB, Candurra NA. Biotechnol. J..

[105] Lee A.M., Pasquato A., Kunz S. (2011). Novel approaches in anti-arenaviral drug development. Virology.

[106] Kolokoltsov A.A., Adhikary S., Garver J., Johnson L., Davey R.A., Vela E.M. (2012). Inhibition of Lassa virus and Ebola virus infection in host cells treated with the kinase inhibitors genistein and tyrphostin. Arch. Virol..

[107] Thomsen A.R., Nansen A., Andreasen S.O., Wodarz D., Christensen J.P. (2000). Host factors influencing viral persistence. Philos. Trans. R. Soc. Lond. B. Biol. Sci..

[108] Zuniga E.I., Hahm B., Oldstone M.B. (2007). Type I interferon during viral infections: Multiple triggers for a multifunctional mediator. Curr. Top. Microbiol. Immunol..

[109] Borrow P., Martínez-Sobrido L., de la Torre J.C. (2010). Inhibition of the type I interferon antiviral response during arenavirus infection. Viruses.

[110] Cuevas C.D., Lavanya M., Wang E., Ross S.R. (2011). Junin virus infects mouse cells and induces innate immune responses. J. Virol..

[111] Baize S., Pannetier D., Faure C., Marianneau P., Marendat I., Georges-Courbot M.C., Deubel V. (2006). Role of interferons in the control of Lassa virus replication in human dendritic cells and macrophages. Microbes Infect..

[112] Martínez-Sobrido L., Zúñiga E.I., Rosario D., García-Sastre A., de la Torre J.C. (2006). Inhibition of the type I interferon response by the nucleoprotein of the prototypic arenavirus lymphocytic choriomeningitis virus. J. Virol..

[113] Martínez-Sobrido L., Giannakas P., Cubitt B., García-Sastre A., de la Torre J.C. (2007). Differential inhibition of type I interferon induction by arenavirus nucleoproteins. J. Virol..

[114] Pythoud C., Rodrigo W.W., Pasqual G., Rothenberger S., Martínez-Sobrido L., de la Torre J.C., Kunz S. (2012). Arenavirus nucleoprotein targets interferon regulatory factor-activating kinase IKK{varepsilon}. J. Virol..

[115] Rodrigo W.W., Ortiz-Riaño E., Pythoud C., Kunz S., de la Torre J.C., Martínez-Sobrido L. (2012). Arenavirus nucleoproteins prevent activation of nuclear factor kappa B. J. Virol..

[116] Fan L., Briese T., Lipkin W.I. (2010). Z proteins of New World arenaviruses bind RIG-I and interfere with type I interferon induction. J. Virol..

[117] Pindel A., Sadler A. (2011). The role of protein kinase R in the interferon response. J. Interferon Cytokine Res..

[118] Montero H., Trujillo-Alonso V. (2011). Stress granules in the viral replication cycle. Viruses.

[119] Linero F.N., Thomas M.G., Boccaccio G.L., Scolaro L.A. (2011). Junin virus infection impairs stress-granule formation in Vero cells treated with arsenite via inhibition of eIF2 alpha phosphorylation. J. Gen. Virol..

[120] Brostrom C.O., Prostko C.R., Kaufman R.J., Brostrom M.A. (1996). Inhibition of translational initiation by activators of the glucose-regulated stress protein and heat shock protein stress response systems. Role of the interferon-inducible double-stranded RNA-activated eukaryotic initiation factor alpha kinase. J. Biol. Chem..

[121] Cuevas C.D., Lavanya M., Wang E., Ross S.R. (2011). Junin virus infects mouse cells and induces innate immune responses. J Virol..

[122] Oliveira-Nascimento L., Massari P., Wetzler L.M. (2012). The Role of TLR2 in Infection and Immunity. Front. Immunol..

[123] Zhou S., Kurt-Jones E.A., Mandell L., Cerny A., Chan M., Golenbock D.T., Finberg R.W. (2005). MyD88 is critical for the development of innate and adaptive immunity during acute lymphocytic choriomeningitis virus infection. Eur. J. Immunol..

[124] Zhou S., Halle A., Kurt-Jones E.A., Cerny A.M., Porpiglia E., Rogers M., Golenbock D.T., Finberg R.W. (2008). Lymphocytic choriomeningitis virus (LCMV) infection of CNS glial cells results in TLR2-MyD88/Mal-dependent inflammatory responses. J. Neuroimmunol..

[125] Zhou S., Cerny A.M., Bowen G., Chan M., Knipe D.M., Kurt-Jones E.A., Finberg R.W. (2010). Discovery of a novel TLR2 signaling inhibitor with anti-viral activity. Antivir. Res..

[126] Hayes M.W., Carrion R., Nunneley J., Medvedev A.E., Salvato M.S., Lukashevich I.S. (2012). Pathogenic Old World arenaviruses inhibit TLR2/Mal-dependent proinflammatory cytokines *in vitro*. J. Virol..

[127] Bieniasz P.D. (2004). Intrinsic immunity: A front-line defense against viral attack. Nat. Immunol..

[128] Tavalai N., Stamminger T. (2008). New insights into the role of the subnuclear structure ND10 for viral infection. Biochim. Biophys. Acta..

[129] Wolf D., Goff S.P. (2008). Host restriction factors blocking retroviral replication. Annu. Rev. Genet..

[130] Hinson E.R., Joshi N.S., Chen J.H., Rahner C., Jung Y.W., Wang X., Kaech S.M., Cresswell P. (2010). Viperin is highly induced in neutrophils and macrophages during acute and chronic lymphocytic choriomeningitis virus infection. J. Immunol..

[131] Sakuma T., Sakurai A., Yasuda J. (2009). Dimerization of tetherin is not essential for its antiviral activity against Lassa and Marburg viruses. PLoS One.

[132] Radoshitzky S.R., Dong L., Chi X., Clester J.C., Retterer C., Spurgers K., Kuhn J.H., Sandwick S., Ruthel G., Kota K. (2010). Infectious Lassa virus, but not filoviruses, is restricted by BST-2/tetherin. J. Virol..

[133] Everett R.D., Chelbi-Alix M.K. (2007). PML and PML nuclear bodies: Implications in antiviral defence. Biochimie.

[134] Bernardi R., Pandolfi P.P. (2007). Structure, dynamics and functions of promyelocytic leukaemia nuclear bodies. Nat. Rev. Mol. Cell Biol..

[135] Borden K.L., Culjkovic B. (2009). Perspectives in PML: A unifying framework for PML function. Front. Biosci..

[136] Lang M., Jegou T., Chung I., Richter K., Münch S., Udvarhelyi A., Cremer C., Hemmerich P., Engelhardt J., Hell S.W. (2010). Three-Dimensional organization of promyelocytic leukemia nuclear bodies. J. Cell Sci..

[137] Dyck J.A., Maul G.G., Miller W.H., Chen J.D., Kakizuka A., Evans R.M. (1994). A novel macromolecular structure is a target of the promyelocyte-retinoic acid receptor oncoprotein. Cell.

[138] Lavau C., Marchio A., Fagioli M., Jansen J., Falini B., Lebon P., Grosveld F., Pandolfi P.P., Pelicci P.G., Dejean A. (1995). The acute promyelocytic leukaemia-associated PML gene is induced by interferon. Oncogene.

[139] Chee A.V., Lopez P., Pandolfi P.P., Roizman B. (2003). Promyelocytic leukemia protein mediates interferon-based anti-herpes simplex virus 1 effects. J. Virol.

[140] Regad T., Chelbi-Alix M.K. (2001). Role and fate of PML nuclear bodies in response to interferon and viral infections. Oncogene.

[141] Borden K.L., Campbell Dwyer E.J., Salvato M.S. (1998). An arenavirus RING (zinc-binding) protein binds the oncoprotein promyelocyte leukemia protein (PML) and relocates PML nuclear bodies to the cytoplasm. J. Virol..

[142] García C.C., Topisirovic I., Djavani M., Borden K.L., Damonte E.B., Salvato M.S. (2010). An antiviral disulfide compound blocks interaction between arenavirus Z protein and cellular promyelocytic leukemia protein. Biochem. Biophys. Res. Commun..

[143] Borden K.L., Campbell Dwyer E.J., Salvato M.S. (1997). The promyelocytic leukemia protein PML has a pro-apoptotic activity mediated through its RING domain. FEBS Lett..

[144] Djavani M., Rodas J., Lukashevich I.S., Horejsh D., Pandolfi P.P., Borden K.L., Salvato M.S. (2001). Role of the promyelocytic leukemia protein PML in the interferon sensitivity of lymphocytic choriomeningitis virus. J. Virol..

[145] Bonilla W.V., Pinschewer D.D., Klenerman P., Rousson V., Gaboli M., Pandolfi P.P., Zinkernagel R.M., Salvato M.S., Hengartner H. (2002). Effects of promyelocytic leukemia protein on virus-host balance. J. Virol..

[146] El McHichi B., Regad T., Maroui M.A., Rodriguez M.S., Aminev A., Gerbaud S., Escriou N., Dianoux L., Chelbi-Alix M.K. (2010). SUMOylation promotes PML degradation during encephalomyocarditis virus infection. J. Virol..

